# Assessment of Bacterial Antibiotic Resistance Transfer in the Gut

**DOI:** 10.1155/2011/312956

**Published:** 2011-01-24

**Authors:** Susanne Schjørring, Karen A. Krogfelt

**Affiliations:** Department of Microbiological Surveillance and Research, Statens Serum Institut, Artillerivej 5, 2300 Copenhagen S, Denmark

## Abstract

We assessed horizontal gene transfer between bacteria in the gastrointestinal (GI) tract. During the last decades, the emergence of antibiotic resistant strains and treatment failures of bacterial infections have increased the public awareness of antibiotic usage. The use of broad spectrum antibiotics creates a selective pressure on the bacterial flora, thus increasing the emergence of multiresistant bacteria, which results in a vicious circle of treatments and emergence of new antibiotic resistant bacteria. The human gastrointestinal tract is a massive reservoir of bacteria with a potential for both receiving and transferring antibiotic resistance genes. The increased use of fermented food products and probiotics, as food supplements and health promoting products containing massive amounts of bacteria acting as either donors and/or recipients of antibiotic resistance genes in the human GI tract, also contributes to the emergence of antibiotic resistant strains. This paper deals with the assessment of antibiotic resistance gene transfer occurring in the gut.

## 1. Emergence of Antibiotic Resistance

The introduction of antibiotics after World War I resulted in a dramatic decrease of numbers of deaths due to bacterial infections. Today, antibiotics have lost their status as the “miracle drug” [1, 2] and “treatment failure” is a new and often seen situation [1–5]. The increase of antibiotic resistance is to be blamed for this medical emergency. The sustainability of antibiotic resistance is partly due to selection of already resistant bacteria that become the new dominant population in the environment. Furthermore, antibiotic usage urges bacteria sensitive to antibiotics to become resistant in order to survive. Survival mechanisms include the acquisition of antibiotic resistance genes from other bacteria/phages (horizontal gene transfer or transduction), mutations in specific genes, and alteration of the bacterial surface. Thus continuous usage and accumulation of antibiotics in the environment has resulted in the increase of antibiotic resistant bacteria not only in Europe but also worldwide. The relationship between antibiotics used as antimicrobial growth promoters (AGPs) in production animals and the development of resistant bacteria in food products has been related to human food born infections with resistant strains. This was not easy to acknowledge. A few countries within the European Union (EU) have acted on the new research regarding the suspicious use of AGP [6]. These countries were Sweden in 1986, Norway in 1995, and Denmark in 1998-1999 [7, 8]. Despite a significant decrease in bacterial antibiotic resistance levels in the countries banning growth promoter products, four different AGPs were in use till January 2006, at which point the EU commission initiated the ban of all AGP [9].

Numerous factors influence the development of antibiotic resistance misuse being the obvious factor. The use of antibiotics is influenced by level of knowledge, expectations, choice of prescription, patient behaviour, economics and health system [10]. Patient-related factors often include inappropriate antibiotic use, like self-medication or inadequate doses despite the prescription text. The prevalence of self-medication in Europe was investigated in 2006 [11]. It was concluded that the levels of self-medication were higher in Eastern and Southern Europe than in Northern and Western Europe. Similarly, Northern countries as well as the Netherlands had the lowest frequency of antibiotic consumption and the lowest level of resistant bacteria [11, 12]. The prescription system for drugs is also important. In developing countries, antibiotics can be purchased in single doses, which increase the risk of the antibiotic treatment being terminated before clinical success. In some countries, antibiotics can be purchased over the counter and prescription is not even necessary, which will also contribute to the rate of incomplete treatments and self-medication. Advertising by television, radio, print media, or internet allows pharmaceutical companies to target a specific brand toward physicians as well as the general public. All of the above-mentioned factors can contribute to a rise in the resistance level [11]. However, further development of antibiotic resistance might be avoided by acquiring knowledge on the mechanisms of bacterial antibiotic resistance. Furthermore, regulatory agencies can set up guidelines and measures, in order to use antibiotics adequately. For example, some countries found that advertising against inappropriate use of antibiotics in national campaigns can reduce the total amount used due to awareness and proper information of the public [13]. During the last decades, there has been an increased focus on persistent bacterial biofilm formation on medical devices, implants, and environmental biofilms [14, 15]. Interestingly, it has been shown that biofilms were hot spot for horizontal gene transfer [16–18]. Thus, promoting development of antibiotic resistance in bacteria. 

Changes in living standards have resulted in a large, ageing human population, and in increased usage of antibiotics. Intensive and long-term hospitalisations due to new advances in medicine often result in new infections (hospital-acquired infections) that are expensive to control and difficult to eradicate. These occur worldwide due to failures in simple infection control, such as inadequate hand hygiene and changing of gloves [19]. Increased usage of broad spectrum antibiotics in order to avoid treatment failure created a vicious circle in the hospitals, as the use of broad spectrum antibiotics influenced the level of multiresistant bacteria and their presence [20]. 

## 2. The Gastrointestinal Tract as a Reservoir of Antibiotic Resistance Genes: Recipients or Donors

The human intestine is a complex ecosystem with a large species diversity, of at least 400 different bacterial species [21, 22]. The density varies through the different parts of the GI tract from 10^4^ bacteria/mL in the stomach to 10^12^ bacteria/g faeces in the distal part of the colon [23, 24]. By classical culture techniques approximately 5–15% of the species present in the GI tract are detected [25]. New estimates by metagenomic approaches of the bacterial flora have suggested that the presence of bacterial species might be as high as 1150 [26]. Despite attempts to stop the antibiotic resistance development, the level of resistant bacteria is on the rise and the hypothesis that our GI tract acts as a reservoir of antibiotic resistance genes is widely accepted [27]. “Could the microflora of the human colon, normally considered innocuous or beneficial, be playing a more sinister role in human health as a reservoir for antibiotic resistance genes?” was the hypothesis set by Salyers and coworkers [28]. 

Furthermore, it is known that genes responsible for antibiotic resistance are present in microorganisms, providing them with self-protection to the antibiotic compounds they produce as defence mechanism against other microorganisms. Similarities among the genes and resistance mechanisms found in the antibiotic producers and in the human pathogenic bacteria suggest that the producer bacteria are the pool of origin of antibiotic resistance genes [29, 30]. 

During antibiotic treatment, all bacteria in the human/animal body are exposed to selective pressure of the antibiotic. Consequently, the GI is highly exposed, especially during oral therapy. This results in the selection of naturally resistant strains carrying an important genetic pool that might be capable of transferring antibiotic resistance genes to other strains present in the human intestine. Moreover, resistant food contaminants that originate from animals and are consumed by humans, can also act as a gene pool (donors) of antibiotic resistance genes [31].

In general, becoming resistance towards antibiotics has been associated with a biological fitness cost. The cost weakens the bacteria's ability to multiply and survive within a host [32]. The connection between resistance and decreased fitness has stimulated the idea that a reduction in the use of antibiotics would lead to a reduction in the frequency of resistant bacteria through natural selection [33]. Furthermore, cross-resistance is of importance. Both* in vitro *and *in vivo *studies have illustrated that “compensatory evolution can stabilise resistant bacterial populations in the absence of antibiotics by making them as fit as susceptible clones” [33]. In addition, resistant bacteria can alleviate the cost of resistance by acquiring additional fitness-compensatory mutations [34–36]. The importance of environmental conditions affecting the fitness costs was also shown [37, 38]. Mutations that have occurred in clinical isolates are seen to compensate for fitness cost in order to stabilise the resistant pathogens in the population [29, 39]. Still, this reversibility in clinical settings is expected to be slow or nonexistent [33].

Resistance genes from both Gram-positive and Gram-negative pathogenic bacteria have revealed almost identical sequences, suggesting that transfer of antibiotic resistance genes across genera has occurred. Furthermore, it is suggested that transfer events have occurred recently and are evolutionary recent events due to high sequence identity [29]. It is also suggested that a gene flux occurs in nature from Gram-positive cocci, (Enterococci/Streptococci) to Gram-negative bacteria [29] with genes coding for streptogramins being described as examples [29]. 

## 3. Effect of Antibiotics to the Gastrointestinal Flora

The physiological effect of the bacterial flora of the GI tract is described as “Fermentation of non-digestible dietary residues and endogenous mucus: salvage of energy as short-chain fatty acids, production of vitamin K, absorption of ions; control of epithelial cell proliferation and differentiation; development and homoeostasis of the immune system and protection against pathogens (the barrier effect)” [40]. A symbiotic relation between the bacteria and the host provides the host with optimal protection. The host provides nutrients to bacteria and bacteria repay by providing a colonisation barrier [40].

When using antibiotics as infection treatment, a disturbance in the flora of the GI tract is created. The barrier is broken and potentially pathogenic bacteria are allowed to colonise the intestine [40]. Disturbances of the normal GI flora are also seen after radical changes of the host diet or after radiation treatments [41]. The effect of different antibiotics on the indigenous flora was investigated and it was found that clindamycin, erythromycin, cefoperazone, ceftriaxone, and moxalactam have a pronounced influence on the flora [41, 42]. The most common complication of antibiotic treatment is excess colonisation of the gut by *Clostridium dificile,* resulting in severe and sometimes fatal enteric diseases [43, 44]. 

Furthermore, antibiotics create a selective pressure on the intestinal flora, risking overgrowth of resistance strains [40]. This, in turn, increases the threat of antibiotic resistant gene transfer among the indigenous flora and, at worst, transfer to other pathogenic bacteria. The risk of developing antibiotic resistant strains which can be transmitted by patient-to-patient contact and the spreading of resistance genes can be diminished by choosing antibiotics with a minimum effect on the GI flora [41]. 

## 4. Model Systems for Studying the Development of Antibiotic Resistance in the Gastrointestinal Tract

In order to assess the effect of antibiotics on the gastrointestinal flora, a number of models have been developed, both in *in vitro* models, where a number of conditions can be controlled as well as *in vivo *animal models, resembling the human/animal host. 

*In vitro* conjugation is conducted in liquid media, on agar plates or on filters placed on agar plates. These are often the first experiments by which frequency of transfer can be observed, since all the parameters can be controlled, that is, growth media, temperature, conjugation time, selective pressure, and so forth. *In vitro* systems trying to mimic the GI tract have been successfully used, such as batch fermentors and continuous flow fermentors, to study the effects of pH on the degradation of nutrients, CO_2_ production among others [45]. 

Different *in vivo* models were used to confirm *in vitro* results, and to mimic the complexity of the host environment. These included alfalfa sprouts as a plant model, and a rumen model, and animal models. [46]. Most experiments related to pathogenic bacteria and their frequency of *in vivo* gene transfer have been conducted in rodents (especially mice). Rodents are often preferred due to high reproduction, short gestation, a minimum of husbandry, and low costs. The histological structure of the GI tract has similarities to the human gut, including epithelial layers and mucous secretion [47]. Effect of bacterial colonisation of the gut, including gene transfer, expression of virulence characteristics, and spatial distribution have therefore been investigated in mice. Since the gut has natural resistance (barrier effect) to colonization by foreign bacterial strains, the experiments are often performed in germ-free and/or antibiotic-treated rodents. Germ-free animals are usually inoculated only with the two strains of interest (donor and recipient strains), thus it is called a diassociated model. The model is a highly controlled setup where only few factors in the GI tract vary. However, a very important factor is that the rodents are coprophages (ingest faeces) which leads to uncontrolled reinoculation. This can be decreased by using gratings and individual cages. The model allows the inoculated bacteria to colonise the GI tract in very high numbers: 10^9^-10^10^ CFU/g faeces. Bacteria which are normally transient in conventional animals will now colonise the GI tract in high numbers due to lack of competition from the indigenous flora [48]. High numbers of the two strains increase the chance of cell-to-cell contact and, therefore, increase the opportunity of gene transfer. Thus, the diassociated model is often described as a worst case scenario model. An investigation of the germ-free mice has revealed that the lymph nodes, spleen, and Peyer's patches are relatively inactive, thus the germ-free mice are immune deficient [49]. 

Another model is the antibiotic-treated mouse model, where the very effective colonising barrier of conventional mice is disturbed by adding streptomycin to the drinking water [50]. Some of the species colonising the gut are eliminated allowing the new bacteria to colonise. When administered orally, antibiotics such as ampicillin, clindamycin, kanamycin, metronidazole, and streptomycin have been shown to be useful in this model [51]. Metronidazole was the least effective antibiotic in decreasing the colonising barrier, whereas clindamycin was active against anaerobic bacteria, kanamycin, and streptomycin against facultative anaerobic bacteria, and ampicillin had a broader range [51]. Hentges et al. found that streptomycin clears off most of the *Enterobacteriaceae*—while the total populations of aerobic and anaerobic bacteria were not affected [52]. Streptomycin increased the level of pH in the GI tracts, enhanced the viscosity of the mucus, making the intestine more susceptible to possible infection by pathogenic bacteria [53, 54]. 

A third model is the human microbiota-associated rodent (HMA) model where germ-free animals are inoculated with human faecal bacteria, thus mimicking the human flora composition in the rodent gut [55]. However, it was shown that changes in the dominant species occasionally occur following inoculation [56]. The shift in dominant species composition may be influenced by the genetic differences between humans and mice, but also by the feed intake. The nutrients present in the GI tract can promote some bacteria and inhibit others [50]. The dominating strain in the human sample might not be the same as the one colonising the animals in high numbers [55, 56]. 

All GI tract animal models have the advantages of small differences in the bacterial flora composition within the animals due to husbandry standards compared to the variance within humans and their individual flora [57]. 

One of the most important points that is worth to discuss concerning the assessment between results obtained from *in vitro *studies, *in vivo* diassociated/conventional animal studies, and extrapolating to humans. The choice of model influences the results and should be addressed carefully. 

*In vitro* and *in vivo* transfer experiments are not comparable and do not always provide the same results and “frequencies”, similarly different types of *in vivo* transfer experiments are not always comparable. The colonising barrier and the complexity of the flora in the antibiotic-treated mice compared to diassociated rats lacking the colonising barrier (as they were germ free before bacterial inoculation) can be considered to be a too simple model, as it does not depict the complexity of the factors involved in transfer of antibiotic resistance genes in the GI tract. Transfer in diassociated animals only provides the knowledge that transfer can occur during colonisation of the GI tract during less realistic circumstances with no indigenous flora present and consequently unusually high CFU levels of both the donor and recipient strains. Differences in biochemistry, physiology, and immunology have been observed when comparing conventional and germ-free animals [58, 59]. The influence of these factors on the bacteria interactions and indirectly on the transfer frequency is still unknown. Using the antibiotic-treated mice model with the conventional bacterial flora barrier and an intact immune system has the advantage of mimicking the human GI tract better. However, a downside of using a model with a complex flora is that gene transfer might occur to other recipient strains than the specific strain which is being investigated, or that the number of transconjugants is below the detection limit (10 CFU/g faeces). The question to be discussed is what is most important in the development of antibiotic resistance—“one” transfer event or the level of transconjugants and their spread.

The level of transconjugants as well as their persistence among the indigenous flora in the GI tract reflects the influence of the spread and therefore, the antibiotic-treated model seems to give a reliable answer. Nevertheless, transfer *in vivo* cannot be calculated from extrapolations of *in vitro* experiments, and humans and animals are different both in flora composition and metabolism [47]. 

## 5. Lactic Acid Bacteria: Intermediate Hosts of Antibiotic Resistance Genes?

One quarter of all food production is estimated to involve microbial fermentation processes by using lactic acid bacterial (LAB) strains [60], for example, sausage, ham, cheese, and dairy products. In addition, probiotics have become available on the market, containing a single strain or a combination of strains. In order to obtain health benefits, live bacteria around 10^11^ CFU/mL should be ingested daily [61]. The proposed problem is that probiotic strains and starter cultures might contain naturally occurring antibiotic resistance genes. From a point of safety, it is necessary to distinguish between intrinsic and acquired resistance genes and most importantly the transferability of these. The increasing level of consumption of fermented foods (more and more products use starter cultures in their production) and an increasing interest in probiotic products result in high daily occurrences of antibiotic resistance genes in the GI tract. The increased level of ingested Gram-positive bacteria has caused new speculations that these bacteria might also contribute to the reservoir of antibiotic resistance genes, and when the right circumstances are present; these genes could be transferred to the indigenous flora. The worst case scenario is that antibiotic resistance genes are transferred to a pathogenic bacterium which could then lead to treatment failure of an infection. Transfer from LAB strains has been documented *in vitro* [62–66], but very few studies have confirmed this antibiotic resistance transfer *in vivo* and it was only observed in the diassociated animal model [62, 64, 66, 67]. Studying the board-host-range conjugative plasmid pAM*β*1, transfer was observed *in vitro* from different donors, for example, *Lactobacillus* spp. (*L. plantarum*, *L. reuteri, L. fermentum, *and *L. murinus*) to other LAB strains [68–72]. In the diassociated model pAM*β*1 has been transferred from *L. reuteri* to * Enterococcus faecalis* [73] and among *Lactococcus lactis *strains [66], but the transfer among the *L. lactis* strain could not be observed in conventional rats [66]. Gram-positive bacteria seem to have a limited influence on the spread of antibiotic resistance in the GI tract, despite the fact that a few multiresistant LAB strains have been detected in weaning piglets [74]. Nevertheless, conclusive documentation of transfer in the GI tract from LAB strains is lacking and therefore more studies need to be carried out. 

## 6. Antibiotic Resistance Transfer to the Indigenous Flora in the Gut

The hypothesis that the indigenous flora can become a reservoir of antibiotic resistance genes remains to this date neither confirmed nor denied. It is suggested that the Gram-negative part of the flora has an increased prospect to obtain antibiotic resistance genes and might act as a reservoir and transfer the resistance gene further to pathogenic bacteria, which might lead to infections with limited treatment possibilities [75]. This is especially true within a hospital setting, where the antibiotics used are often aimed at the Gram-negative pathogens, adding selective pressure on the Gram-negative bacteria. The human microbiota associated rat model revealed that broad-host range plasmid pAM*β*1 can be transferred from *L. lactis* to indigenous *Enterococcus *spp*., *whereas no transfer was observed to *Lactobacillus* spp., *Bifidobacterium* spp., and *Enterobacteriaceae* spp. [76]. A study using voluntary human subjects in 1974 showed that antibiotic resistance transfer occurred between an *Escherichia coli* of animal origin and indigenous *E. coli* in the GI tract during tetracycline treatment. Transfer was observed late in the experiment (36 days after the end of the treatment), but only with the therapeutic levels of tetracycline (1000 mg/daily) administrated and not with the low level of 50 mg/day [77]. The observation of “late transfer” has also been observed in a study using mice, where the indigenous *E. coli* transconjugant was observed in high numbers 23 days after inoculation of the multiresistant* Klebsiella pneumoniae* donor [75]. In addition, vancomycin resistance has been observed to be transferred between *Enterococcus* spp. in the GI of humans [78]. Furthermore, the possible transfer of a plasmid harbouring ampicillin resistance from the food contaminant *Salmonella Enteritidis* to indigenous *E. coli* in the GI tract of humans was described, and *in vitro* experiments confirmed that transfer was possible at high frequencies [79]. Transfer of any antibiotic resistance genes is a general concern, yet transfer of Extended spectrum beta-lactamases (ESBL) resistance genes is a category of its own, which might result in treatment limitations and in worst cases of treatment failure [4, 80]. Interestingly, indigenous *E. coli* strains with a *bla *_CTX-M_ ESBL gene were isolated form pigs. During treatment with cephalosporin's (veterinary treatment), the diversity of the indigenous* E. coli* strains receiving the *bla *_CTX-M_ genes increased, suggesting horizontal gene transfer during selective pressure [81]. If meat for consumption is contaminated with the faecal ESBL producing *E. coli, *and if the meat is not properly processed, humans can ingest the ESBL producing *E. coli *and the gene transfer can occur again in the human GI tract. An example of selective pressure increasing the possibility of transfer was described by a clinical case documenting transfer of ACC-1 AmpC (ESBL) from *K. pneumoniae* to indigenous *E. coli* during antibiotic treatment of 1-year old boy [82]. The child was colonised with a *K. pneumoniae* harbouring ACC-1 AmpC. The postoperative treatment was imipenem and amikacin which lead to a spontaneous mutation that decreased the sensitivity towards imipenem. Subsequently, the plasmid was transferred to a commensal ampicillin resistant *E. coli* during treatment with cefotaxim due to urinary tract infection caused by the *E. coli* [82]. This is a very important case story illustrating and emphasising the complexity of circumstances and mechanisms during antibiotic resistance gene transfer in the gut. 

## 7. Farm-to-Fork Perspective

**Figure 1:**
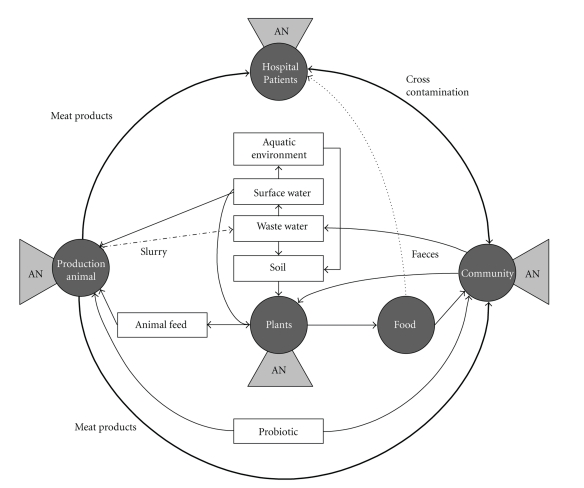
Schematic representation of the complexity of selection/development of antibiotic resistant bacteria in different known reservoirs. The possible routes of transmission throughout the environment of these resistant bacteria are suggested. The reservoirs where antibiotics are applied are also suggested as hot spots for horizontal gene transfer. AN: antibiotic treatment/pest control, adapted from [83].

The ecological niches of human, animal, water, and soil can easily be evaluated as separated small niches, but this evaluation might not yield the entire truth. Bacteria are present in microecological niches, but move between ecosystems from animals to humans, from humans and animals (faeces and manure) to water and soil and return to human and animals, through, for example, food (plants or vegetables). In addition, the use of antibiotic treatment in each small niche (humans, animals or plants) selects the resistant strains to become the reservoir of resistance genes. These antibiotic resistance genes are present and can be transported within the bacteria from one niche to another.  Figure 1 illustrates the interaction between the different reservoirs of bacteria in the food chain [83]. The nonpathogenic bacteria are widely used as feed additives of animal feed, as starter cultures in food preparation, or as probiotics for humans. Antibiotic treatment of humans (in the hospitals or in the community), animals, and pest control on plants, creates a selection of resistant bacteria in those areas. Humans ingest meat and plants which might contain bacteria with antibiotic resistance genes, but the increasing consumption of fermented food products increases the risk of antibiotic resistance genes to occur in the intestine. It is a well-known fact that human foodstuffs contain bacteria harbouring antibiotic resistance genes, but the main focus has been on pathogen contaminants in foods. Raw food, which is not properly processed before eating, might result in infections caused by pathogenic resistant bacterial strains. 

Epidemiologically studies have shown that through trace back, an outbreak strain can be related to a specific food product [84], for example, the presence of multiresistant *Salmonella typhimurium* in Carpaccio [85], *Shigella sonnei* infections caused by imported baby corn [86], *E. coli* O157 infections caused by contaminated lettuce [87], and a recent outbreak caused by *Salmonella kedougou* [88]. However, antibiotic resistant pathogenic strains are also frequently detected in production animals in Europe, such as MRSA in pigs [89], multiresistant *Salmonella infantis* in broiler chicken [90], ESBL producing *Salmonella *spp. in poultry [91], and also in food products, such as vancomycin resistant *E. faecalis* in turkey meat [92], multiresistant *E. coli* in minced beef [93], and tetracycline resistant *E. faecalis* in poultry meat (chicken, turkey and duck), beef, and pork [94].

It has also been demonstrated that antibiotic resistance genes of different Lactic acid bacterial species, for example, *Lactococcus* spp., *Streptococcus thermophilus,* and *Pseudomonas *spp., are present in “ready to use” products such as different cheese products, raw milk, meat products like pork chop, turkey, and beef as well as in mushroom and spinach [95]. *In vitro* experiments have shown that *L. lactis* DNA, containing the tetracycline resistance gene *tet*S, was successfully transformed into the oral cariogenic pathogen *Streptococcus mutans* [95]. Although this experiment illustrates the transfer of antibiotic resistant genes from a commensal to a pathogenic bacterium, these events have not been confirmed *in vivo*. Other studies showed transfer of erythromycin resistance gene *erm*B from a commensal *L. plantarum* strain to *E. faecalis in vivo* in the diassociated rat model [62]. However, this was not reproducible in the streptomycin-treated mice model [62]. Although probiotic and industrial bacterial cultures were seen to possess the acquired antibiotic resistance genes, no transfer *in vitro* was observed, neither intraspecies nor interspecies [96]. Whereas few natural resistant LAB were isolated from cheese (raw milk without starter cultures), only two *L. lactis* strains harbouring the conjugative *tet*M resistance transposon Tn916 were able to transfer to both *Lactococcus* and *Enterococcus* [97]. 

Transfer of antibiotic resistance genes from Gram-positive to Gram-negative bacteria *in vitro* is a very rare event. I*n vitro* transfer of a naturally occurring Gram-positive plasmid pIP501 in *E. faecalis* to *E. coli* has been described [98]. Another naturally occurring plasmid, pIP823 from *Listeria monocytogenes,* was only transferred in the presence of the broad host range plasmid, pAM*β*1 which mobilised the transfer to both *E. faecalis* and *E. coli *[99]. Nevertheless, sequence analysis documents almost identical antibiotic resistance genes and thereby suggests that transfer occur from Gram-positive to Gram-negative bacteria strains [29]. In addition, the opposite transfer situation is not believed to be possible because of the difference in the gene expression systems [29]. 

Plasmid transfer across genus from LAB have been rarely described and few studies are performed in an *in vivo* model like the diassociated rats [62, 73]. The many *in vitro* studies showed that the Gram-negative flora harbours the mobilised plasmids and the main focus in antibiotic resistance development should remain on these. Furthermore, transposons like the Tn916 (harboring tetracycline resistance) were also shown as being transferable *in vitro* between Gram-positive and Gram-negative genus [100]. Studies like this *in vitro *transfer event should be further investigated in *in vivo* models.

Transgenic food has been investigated for its influence on the spread of antibiotic resistance, and the conclusion is that there is very little reason to assume that consumption of transgenic food or feed additives increases the risk. Different environmental factors have an impact on HGT events, selective advantage for the bacterial population being the most important [101]. 

## 8. Concluding Remarks

A greatly respected scientist within the antibiotic resistance area has recently stated that “Evolution of bacteria towards antibiotic resistance is unavoidable as it represents a particular aspect of the general evolution of bacteria. Thus, at the very best, the only hope we can have in the field of antibiotic resistance is to delay dissemination of resistant bacteria or resistance genes” [29]. In addition, it is suggested that identifying more resistance mechanisms in antibiotics producing strains might be the solution to predict the mechanisms that will be observed in the human pathogenic strains in the future [29]. However, horizontal gene transfer does not appear to have homogenised the bacteria. Genetic diversity and a well-defined phylogenetic tree for bacteria are still the rule rather than the exception [102]. 

High level of antibiotic resistance, combined with the tendency to treat infections with more broad-spectrum drugs, results in a selection of multiresistant bacteria. Due to import/export of food products and travelling habits, a single part of the world cannot deal with this threat alone. The optimal solution would be worldwide consensus on the use of antibiotics, both in health care and in the veterinary sector. 

There is no doubt that antibiotic treatment is a necessity, and the influence on the total GI flora is a matter of secondary importance. Conversely, antibiotic treatment creates a great advantage for resistant bacteria which is selected to colonise the intestine and treatment might result in antibiotic resistance transfer among Gram-negative bacteria, or to the indigenous flora or even a pathogen. The ecological effects of antibiotic treatment on the commensal microflora should be the focus of more studies in the future. Rational use of antibiotics together with infection control will possibly limit further spread of multiresistant bacteria, but no matter what boundaries we set up, transfer of antibiotic resistance genes among bacteria can and will occur.
